# Prioritizing Chemicals and Data Requirements for Screening-Level Exposure and Risk Assessment

**DOI:** 10.1289/ehp.1205355

**Published:** 2012-09-20

**Authors:** Jon A. Arnot, Trevor N. Brown, Frank Wania, Knut Breivik, Michael S. McLachlan

**Affiliations:** 1 Department of Physical and Environmental Sciences, University of Toronto Scarborough, Toronto, Ontario, Canada; 2 Norwegian Institute for Air Research, Kjeller, Norway; 3 Department of Chemistry, University of Oslo, Oslo, Norway; 4 Department of Applied Environmental Science (ITM), Stockholm University, Stockholm, Sweden

**Keywords:** exposure, high throughput, organic chemicals, risk, uncertainty analysis

## Abstract

Background: Scientists and regulatory agencies strive to identify chemicals that may cause harmful effects to humans and the environment; however, prioritization is challenging because of the large number of chemicals requiring evaluation and limited data and resources.

Objectives: We aimed to prioritize chemicals for exposure and exposure potential and obtain a quantitative perspective on research needs to better address uncertainty in screening assessments.

Methods: We used a multimedia mass balance model to prioritize > 12,000 organic chemicals using four far-field human exposure metrics. The propagation of variance (uncertainty) in key chemical information used as model input for calculating exposure metrics was quantified.

Results: Modeled human concentrations and intake rates span approximately 17 and 15 orders of magnitude, respectively. Estimates of exposure potential using human concentrations and a unit emission rate span approximately 13 orders of magnitude, and intake fractions span 7 orders of magnitude. The actual chemical emission rate contributes the greatest variance (uncertainty) in exposure estimates. The human biotransformation half-life is the second greatest source of uncertainty in estimated concentrations. In general, biotransformation and biodegradation half-lives are greater sources of uncertainty in modeled exposure and exposure potential than chemical partition coefficients.

Conclusions: Mechanistic exposure modeling is suitable for screening and prioritizing large numbers of chemicals. By including uncertainty analysis and uncertainty in chemical information in the exposure estimates, these methods can help identify and address the important sources of uncertainty in human exposure and risk assessment in a systematic manner.

The primary objective of chemical assessment programs is to identify and regulate chemicals that may cause harmful effects to humans and ecosystems. Tens of thousands of chemicals require evaluation; however, data and resources are limited (Muir and Howard 2006). In particular, monitoring data that could be used in exposure assessment are available for only 1–2% of the chemicals for which there are at least some toxicity data (Egeghy et al. 2012). The Canadian Domestic Substances List categorization highlighted that of the approximately 11,300 listed organic chemicals merely 3% have measured bioaccumulation data (only in fish), 4% have measured half-lives in air (based on laboratory simulated reactions), and only 12 chemicals have measured biodegradation half-lives in water, soil, or sediment (Arnot and Gobas 2006; Environment Canada 2006). Furthermore, it would be impossible to measure all chemicals in all media to which humans and ecological receptors are exposed. These data gaps necessitate the development and application of conceptual, mass balance, and quantitative structure–activity (property) relationship [QSA(P)R] models. Because there are so many chemicals requiring assessment and such extensive data gaps, it is difficult to determine which chemicals pose the greatest exposure and risk and what chemical information contributes the greatest uncertainty in these assessments.

Methods to screen and prioritize chemicals for more comprehensive evaluations include “separate” persistence, bioaccumulation, and toxicity (PBT) classification categories and “holistic” multimedia, multipathway mass balance exposure and risk assessment models that simulate key processes in the source–receptor relationship. The PBT method employs multiple bright-line pass/fail criteria and mass balance and QSA(P)R models for data generation. The pass/fail criteria are variable (i.e., dependent on the regulatory program) and the multiple binary scoring results reduce consistency and transparency in decision making (Arnot and Mackay 2008; van Wezel and Jager 2002). Although uncertainty is prevalent because of data gaps and the necessary reliance on models (Arnot and Gobas 2006; Zhang et al. 2010), the PBT pass/fail methods do not provide guidance for addressing uncertainty. In contrast, “holistic” mass balance exposure models provide single numerical values for screening and priority setting [e.g., chemical intake fraction (Bennett et al. 2002)], and they can also include sensitivity and uncertainty analyses (Hertwich et al. 1999; Huijbregts et al. 2000; McKone 1994) using the same basic chemical information included in PBT methods (i.e., partitioning and degradation rate data). Exposure models also provide the opportunity to better understand key mechanistic processes in the source–receptor relationship and the model predictions (hypothesis based) can be evaluated with monitoring data (test derived) (Cowan-Ellsberry et al. 2009; Sheldon and Cohen Hubal 2009). Finally, although the actual chemical emission rate clearly influences exposure and risk, it is not directly included in a PBT assessment.

In this article, we describe the parameterization and application of a mass balance model to screen and prioritize > 12,000 organic chemicals using four far-field human exposure and human exposure potential assessment metrics. The propagation of uncertainty in model input parameters is included through the model calculations, thus providing uncertainty estimates for the four assessment metrics. A primary objective was to use these results as a case study to obtain a better quantitative perspective of the relative uncertainties in chemical information required for screening-level exposure and risk assessment. In addition, we consider external and internal human exposure metrics and discuss recommendations for future research needs to improve assessments.

## Methods

**Figure 1:**
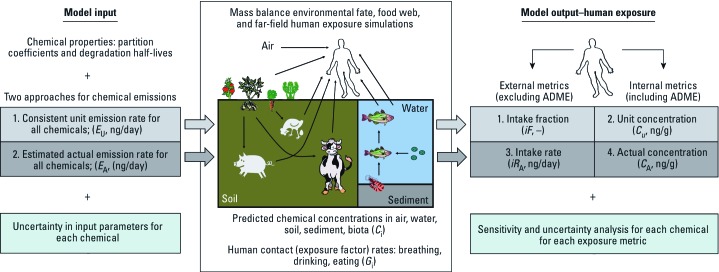
Schematic of the far-field human exposure model simulations highlighting the two human exposure potential metrics (light gray boxes, calculated using unit emission rates) and the two actual human exposure metrics (dark gray boxes, calculated using actual emission rate estimates); ADME, absorption, distribution, metabolism, excretion processes.

*Multimedia, multipathway far-field human exposure models and metrics*. Figure 1 illustrates far-field human exposure concepts and four assessment metrics. Far-field exposures are the result of human contact with chemicals in outdoor air, drinking water, and food as a result of general chemical use and release throughout the chemical life cycle and subsequent chemical fate and transport in the physical environment (air, water, soil, and sediment) and food web bioaccumulation. In the present study, we assumed that there were no losses or additions of chemical to food as a result of processing and preparation (e.g., washing, packaging, cooking) and that all food sources originate in the same regional environment in which the human resides. Near-field exposures such as dermal, indoor, occupational, industrial, and direct exposure pathways from consumer use (e.g., application of personal care products) were not considered in this case study.

Various studies have proposed metrics for assessing human exposure and human exposure potential (e.g., Arnot et al. 2010; Bennett et al. 2002; Cowan-Ellsberry et al. 2009). Exposure metrics based on actual chemical emission rates (*E*_A_; kilograms per hour or nanograms per day) provide actual exposure estimates such as chemical concentrations in humans (*C*_A_; nanograms per gram) and human intake rates (*iR*_A_; nanograms per day). For each exposure pathway (i.e., air, food, water), the intake rate is the product of the environmental medium intake, or contact rate (e.g., inhalation rate, *G*_i_; grams per day), and the chemical concentration in the corresponding medium (e.g., concentration in air, *C*_i_; nanograms per gram). Aggregate exposures are the sum of all of the exposure pathways considered in the assessment. Intake rates do not account for chemical absorption, distribution, metabolism, and excretion (ADME) processes in the receptor of interest; however, internal concentrations do account for ADME processes. Actual exposure estimates are applicable in risk-based chemical assessments by comparing concentrations or intake rates with concentrations or rates of intake associated with effect or no-effect levels; however, it is often difficult to obtain reliable actual emission rate information. A consistent, arbitrary unit emission rate (*E*_U_; kilograms per hour or nanograms per day) for all chemicals can be used to provide estimates of relative exposure potential for screening and prioritizing chemicals in a hazard-based context. The chemical intake fraction (*iF*; dimensionless, or nanogram-intake/nanogram-emission) and the unit emission rate–based concentration (*C*_U_; nanograms per gram) are examples of relative exposure potential metrics that are independent of the actual chemical emission rate. We selected the *iF*, *iR*_A_, *C*_U_, and *C*_A_ metrics to consider a range of possible far-field human exposure assessment objectives.

Various multimedia, multipathway, mass balance exposure models can be used to calculate some of these far-field human exposure metrics (e.g., Arnot and Mackay 2008; Czub and McLachlan 2004; Huijbregts et al. 2000; van Wezel and Jager 2002). We used the Risk Assessment IDentification And Ranking (RAIDAR) version 2.0 model (Arnot and Mackay 2008) because it calculates all four exposure metrics of interest and it allows for the inclusion of chemical-specific biotransformation rate information for vertebrate species, a process that has been shown to strongly affect exposures (Arnot et al. 2010; McLachlan et al. 2011). The primary objective of RAIDAR is to provide a consistent evaluative framework for risk and exposure information for priority setting objectives and for comparative chemical assessments. The model is described in detail elsewhere (Arnot and Mackay 2008; Arnot et al. 2010). Briefly, RAIDAR combines user-supplied information on chemical emissions and properties with a mechanistic description of chemical phase distribution, intermedia transport, and degradation processes to calculate concentrations in air, water, soil, and sediment of a generic regional-scale (100,000 km^2^) environment. Using these concentrations in the physical environment, RAIDAR calculates bioaccumulation in aquatic, terrestrial, and agricultural food webs to estimate exposures of, and potential risks to, humans and representative ecological species (e.g., plants, invertebrates, fish, birds, and mammals). The model assumes chemical emissions are continuous from diffuse, nonpoint sources, and losses from the regional environment can include reaction (i.e., degradation in environmental media) and advection (e.g., outflow in air and water, burial in sediment). Primary producers and invertebrates bioconcentrate chemicals from their ambient environment of air, water, soil, or sediment, whereas vertebrates bioaccumulate chemicals from their ambient environment and from their diet. The bioaccumulation models consist of a mass balance equation formulated over a single compartment for each vertebrate species (including humans) accounting for major processes of chemical uptake (i.e., respiration, drinking water, and dietary exposures for potential biomagnification) and elimination (e.g., respiration, fecal egestion, urinary excretion, and biotransformation). RAIDAR calculates concentrations in outdoor air, water, soil, sediment, and biota, including humans, using either *E*_U_ or *E*_A_ for a particular simulation. For this case study, RAIDAR version 2.0 was coded in Visual Basic for Applications hosted in Excel (Microsoft Corp., Redmond, WA, USA) to quantify the propagation of uncertainty in chemical properties (model input parameters) in the calculated *iF*, *iR*_A_, *C*_U_, and *C*_A_ exposure metrics (i.e., model output).

*Case study chemicals*. We compiled a database of 12,619 organic chemicals including organic chemical substances with reported production in Europe, the United States, Canada, Japan, and other countries that participate in the Organisation for Economic Co-operation and Development. This database comprises a broad range of chemical properties and production volumes and is considered to represent much of the diversity of current-use organics [for details, see Supplemental Material, p. S2 (http://dx.doi.org/10.1289/ehp.1205355)].

*Model parameterization and high throughput screening applications*. The RAIDAR model input parameters required for assessing exposure potential are molar mass (*M*; grams per mole), octanol–water partition coefficient (*K*_OW_; dimensionless), Henry’s law constant (*H*; pascal.cubic meter/mole), degradation half-lives (*HL*s; hours) in air, water, soil, and sediment and primary biotransformation *HL*s in vertebrates. The dimensionless air–water partition coefficient (*K*_AW_) is calculated by dividing *H* by the gas law constant (*R*; 8.314 Pa.m^3^/mol.K) and absolute system temperature (*T*; e.g., 298 K). The model uses the octanol–air partition coefficient (*K*_OA_; dimensionless) for processes such as aerosol–air partitioning and bioaccumulation in air-breathing organisms; however, *K*_OA_ is not a required input parameter because it is calculated internally by the model as *K*_OW_/*K*_AW_. Chemical mode-of-entry information is required (i.e., relative percent release of a chemical to air, water, and soil) for Level III (i.e., steady state, nonequilibrium) fate simulations. For assessments of actual exposure, an estimate of the regional-scale actual emission rate (*E*_A_; e.g., kilotonnes/year) is also required.

Table 1 summarizes the range and median values for selected model input parameters. A valuable source of information for obtaining chemical partitioning properties and reaction half-lives for chemical screening is the U.S. Environmental Protection Agency’s (EPA) Estimation Program Interface Suite (EPI Suite™) software program (U.S. EPA 2011). The software is free and publicly available and requires only chemical structural information [i.e., simplified molecular input line entry system (SMILES) notations (Weininger 1988)] for searching large databases of measured information and generating QSA(P)R predictions. We selected measured values for chemical properties preferentially over QSA(P)R estimates. There are technical and analytical challenges associated with accurately measuring certain chemical properties (i.e., substances with low vapor pressure or low water solubility). For some chemicals, the QSA(P)R-predicted properties are beyond the domain of measured values used to develop and test the QSA(P)Rs. Although it is possible for properties to exist beyond the range of currently measured domains, these QSA(P)R predictions may have substantial errors. As a part of this case study, we identified and counted predicted partitioning properties outside of the current measurement domains. We replaced predicted properties outside of the measured domains with selected measured “maxima” or “minima” values for these simulations. For ionogenic organics, EPI Suite™ version 4.1 (U.S. EPA 2011) provides property estimates for the neutral species only. We did not consider dissociation in this case study because of a general lack of data (e.g., p*K*as) and publicly available high throughput models for parameterizing diverse types of ionogenic chemicals. Further details on the selection of partitioning properties are in the Supplemental Material, pp. S2–S4 (http://dx.doi.org/10.1289/ehp.1205355). Further details on the selection of degradation half-lives are in the Supplemental Material, pp. S4–S7.

**Table 1:** Summary of model input parameters and associated Cfs [ranges (medians)] for 12,619 organic substances.

Model input parameter	Input parameters	Cf for model input parameters
M (g/mol)	16–1,580 (240)	NA
KAW (dimensionless)	10–12–103 (1.2 × 10–6)	6–300 (100)
KOW (dimensionless)	10–4–109 (1.3 × 103)	3–30 (10)
HL–air (hr)	4.7 × 10–4–1.3 × 106 (4.8)	10–100 (10)
HL–water (hr)	12–9.0 × 104 (570)	11–3,200 (14)
HL–soil (hr)	23–1.8 × 105 (1,100)	22–6,400 (27)
HL–sediment (hr)	100–8.1 × 105 (5,200)	33–9,700 (40)
HLBIO–fish (hr)	0.42–6.0 × 1011 (16)	10–15 (15)
HLBIO–avian/mammalian (hr)	0.42–6.0 × 1011 (16)	10–45 (45)
EA (kt/year)	5.1 × 10–9–180 (4.8 × 10–5)	71–10,000 (500)
Abbreviations: Cf, confidence factor; HLBIO, biotransformation half-life; NA, not applicable.				

We used a unit emission rate (*E*_U_) of 1 kg/hr for all chemicals to estimate human exposure potential (e.g., *iF*, *C*_U_). Estimated “actual” regional-scale emission rates (*E*_A_) are needed for estimates of actual exposures and Level III fate calculations require mode-of-entry information. We used production volume estimates and the European Union Technical Guidance Document (EU TGD) emission factor scenarios (European Commission 2003) to estimate *E*_A_ and chemical mode-of-entry to the environment [see Supplemental Material, pp. S7–S8 (http://dx.doi.org/10.1289/ehp.1205355)]. The EU TGD emission factors (Supplemental Material, Table S1) represent “default” recommended values and are assumed to provide information for the relative release of a chemical to air, water, and soil using physical–chemical properties alone. We assumed that the chemicals are used widely and that advective loss from one regional environment is compensated for by advective inflow from a neighboring region. The advective flow residence times in air and water were set to 10^11^ hr to parameterize the model to satisfy this assumption.

*Sensitivity and uncertainty analysis*. Exposure estimates are subject to uncertainty whether the data are measured or modeled. We used an analytical method to estimate uncertainty in our modeled exposure data because of its relative simplicity, and because of the extensive data gaps, the uncertainty in most of the chemical parameters can only be approximated using QSA(P)R models and expert judgment (MacLeod et al. 2002; Slob 1994). The sensitivity of a model input parameter quantifies the change in model output as a function of a defined change in the model input parameter. For example, the sensitivity (*S*) of an input parameter (*P*_i_), such as *K*_OW_, on model output, such as *iF*, can be approximated as

*S* = (Δ*iF*/*iF*)/(Δ*P*/*P*_i_), [1]

where Δ*iF* is the change in the *iF* value and Δ*P* is a fixed change to a selected input parameter value (e.g., 0.1% change in *K*_OW_). The contribution to variance (*CV*; uncertainty) of the chemical input parameters on an exposure calculation for that particular chemical (*CV*_i_) can be evaluated as a function of the variance (uncertainty) σ^2^_i_ and sensitivity (*S*_i_) of the individual input parameters (*P*_i_s) for all model input parameters (*n*) for that particular chemical as


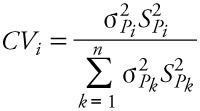
[2]

We used confidence factors (*Cf*), also referred to as distribution factors (Slob 1994), to quantify uncertainty (variance) in model input parameters. The *Cf* is a readily interpretable expression of the variance in a log-normally distributed parameter. A *C*ƒ of 10 suggests that 95% of all of the values in the distribution are within 10 and 0.1 times the median value. A 95% probability for a log-normally distributed parameter *X* with a median *M* is expressed as described by MacLeod et al. (2002) and Slob (1994) as


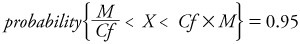
[3]

Thus, the variance in the log-normal distribution increases with an increase in *C*ƒ. The use of *C*ƒs “is particularly useful when data are scarce and the magnitude of the uncertainty can only be roughly quantified by giving approximate lower and upper bounds using expert judgment” (Slob 1994). *C*ƒs can be calculated from estimates of variance such as SDs for log-transformed, lognormal distributions (σ_log_*_X_*; base 10 logarithm) as described by MacLeod et al. (2002) and Slob (1994) as

*C*ƒ = *e*^[1.96 σ_log_^*^_X_^* ^ln(10)]^. [4]

We assumed model input parameters to be log-normally distributed, and because of data gaps, we assigned *C*ƒs using professional judgment. We used SDs from the EPI Suite™ (U.S. EPA 2011) QSA(P)R training and testing sets to guide the application of professional judgment for calculating and assigning screening-level *Cf*s using Equation 4. In general, the relative uncertainty in chemical information progresses as follows: measured data < predicted data “within the defined domain” < predicted data “outside the defined domain.” Table 1 summarizes the ranges and medians for *Cf*s for model input parameter categories [for further details, see Supplemental Material, pp.S2–S8 (http://dx.doi.org/10.1289/ehp.1205355)].

## Results

**Figure 2:**
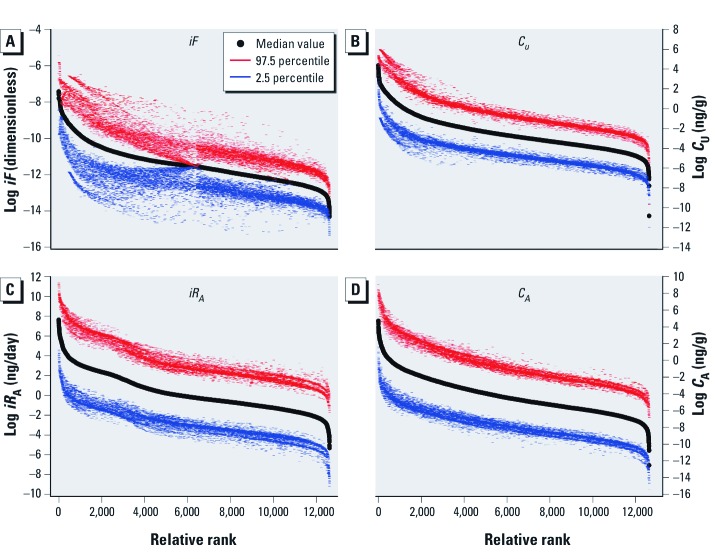
Relative ranking of 12,619 organic substances for far-field model estimates of *iF*s (*A*), *C*_U_s (*B*), *iR*_A_s (*C*), and *C*_A_s (*D*). Note that the relative rankings (*x*-axis) are unique for each metric.

Figure 2 illustrates the screening and ranking of 12,619 organic substances based on four far-field exposure and exposure potential metrics. Figure 2C and D show results using estimates of actual emission rates (i.e., *iR*_A_ and *C*_A_) and Figure 2A and B show results using a consistent unit emission rate for all chemicals (i.e., *iF* and *C*_U_). The upper tails of the rankings suggest that all methods have the capacity to differentiate chemicals with relatively higher exposure and exposure potential from all chemicals in the database; however, the actual exposure metrics show larger ranges than the comparative exposure potential metrics because of the greater range of possible *E*_A_ values. Estimates of actual exposures span approximately 15 and 17 orders of magnitude for *iR*_A_ and *C*_A_, respectively. Estimates of exposure potential span approximately 7 and 13 orders of magnitude for *iF* and *C*_U_, respectively.

**Figure 3:**
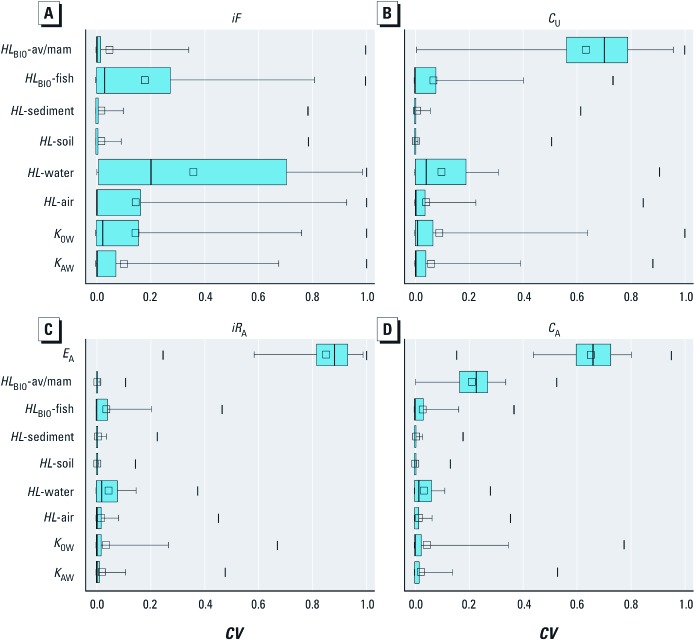
Statistical summary for the *CV* of model input parameters on the calculated far-field human *iF*s, *C*_U_s, *iR*_A_s, and *C*_A_s for the 12,619 substances; *HL*_BIO_–av/mam, *HL*_BIO_–avian/mammalian. Model input parameters are summarized in Table 1. Boxes represent 25th–75th percentiles and whiskers 5th–95th percentiles, vertical lines (|) indicate ranges, lines within boxes are medians, and small squares are means.

Figure 3 provides a statistical summary of *CV* for modeled exposure metrics (output) as a function of model input parameters for all chemicals. Clearly the greatest contribution to uncertainty in estimates of actual internal (Figure 3D) and external (Figure 3C) human exposure is the estimate of *E*_A_. The uncertainty in *E*_A_ is particularly dominant for the *iR*_A_ calculations. These results also reflect that the sensitivity of the model exposure calculations to the actual emission rate is 1. In other words, the response of the model is linear for this input parameter, so a 2-fold change in *E*_A_ results in a 2-fold change in *iR*_A_ and *C*_A_. In general, the primary biotransformation *HL* (*HL*_BIO_) in mammals is the input parameter with the second greatest contribution to variance in *C*_A_ calculations. This is because the primary biotransformation *HL* in the human is a key determinant of the overall residence time in the body for many parent chemicals. Chemicals with log *K*_OW_ > 2 and log *K*_OA_ > 6 have high bioaccumulation potential in humans when biotransformation is assumed to be negligible (Czub and McLachlan 2004; Kelly et al. 2007), and biotransformation is a key factor for reducing bioaccumulation potential over a wide range of chemical partitioning properties (McLachlan et al. 2011). For more water-soluble and volatile chemicals, urinary excretion and respiration can be relatively quicker routes of elimination; thus for these types of chemicals, *HL*_BIO_ is not as important in the calculation of *C*_A_. *iR*_A_ does not consider absorption, biotransformation, or elimination in humans; however, in the *iR_A_* calculation, these processes are considered in vertebrates that are part of the agricultural food web and thus part of the human diet.

Figure 3A and B refer to unit emission metrics of human exposure potential (i.e., independent of *E*_A_). For *iF* simulations, a number of input parameters contribute to variance including (in general order of magnitude) degradation *HL* in water, biotransformation *HL* in fish, degradation *HL* in air, *K*_OW_, and *K*_AW_. For certain chemicals the *CV* on *iF* can be 1 for most input parameters (as noted by the maxima points), the only exceptions being for the degradation *HL*s in soil and sediment. These two input parameters are generally shown to have low *CV* in human exposures. In general, *K*_OW_ is shown to have a greater *CV* in exposure metrics than *K*_AW_ despite a relatively greater degree of uncertainty in *K*_AW_ than in *K*_OW_ (Table 1). For *C*_U_, *HL*_BIO_-mammal is generally the greatest source of uncertainty in the calculation. For the same reasons discussed earlier for *C*_A_, the calculation of *C*_U_ is also often sensitive to the biotransformation *HL* input parameter. In general terms, the degradation *HL* in water, the biotransformation *HL* in fish and *K*_OW_ are other parameters contributing the most variance in *C*_U_ calculations.

**Figure 4:**
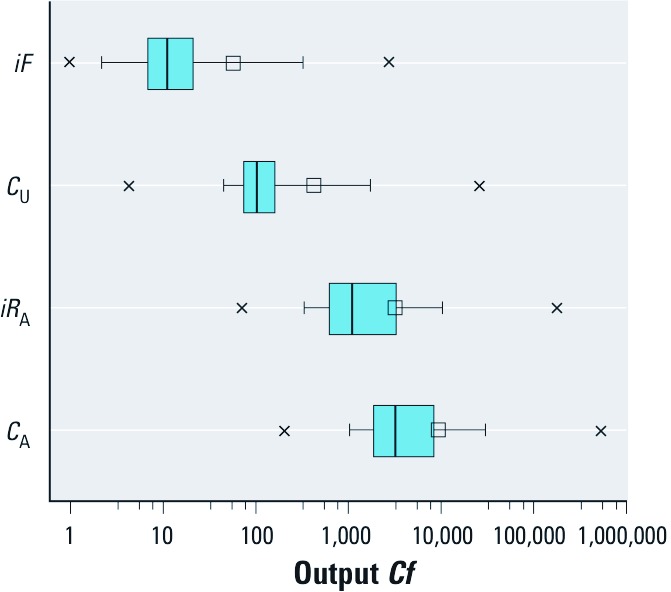
Statistical summary of the modeled output *Cf*s for far-field human *iF*s, *C*_U_s, *iR*_A_s, and *C*_A_s for the 12,619 substances. Boxes represent 25th–75th percentiles and whiskers 5th–95th percentiles, crosses (x) indicate ranges, lines within boxes are medians, and small squares are means.

Figure 4 summarizes the model output *Cf*s for the four exposure metrics. The general trend of relative uncertainty in model calculations is *C*_A_ > *iR*_A_ > *C*_U_ > *iF*. This order predominantly reflects the greater level of uncertainty associated with estimating exposure rather than exposure potential as a result of the high uncertainties in *E*_A_. Calculations for *C*_A_ are generally more uncertain than calculations for *iR*_A_ because of the additional uncertainty associated with biotransformation *HL*s in humans. On average the range associated with a 95% probability in *C*_A_ covers 8 orders of magnitude (Figure 2D). By comparison, on average the range associated with a 95% probability in *iF* covers about 3–4 orders of magnitude (Figure 2A). Approximately one-third of the chemicals in the case study have predicted physical-chemical partitioning property estimates that are outside of the selected domains.

## Discussion

*Chemical screening and prioritization*. Regulatory and scientific agencies such as the U.S. EPA and the National Research Council recognize the need to develop, apply, and evaluate a systems approach that fully integrates exposure and toxicity information in a holistic framework for risk assessment (Cohen Hubal 2009). Such an approach is envisioned to identify and reduce uncertainties in current risk assessment approaches (Sheldon and Cohen Hubal 2009). We compiled the chemical data required to screen and evaluate more than 12,000 organic chemicals using four metrics for far-field human exposure and exposure potential calculated with a mass balance multimedia model and included the propagation of uncertainty in chemical property data on exposure estimates. The mass balance modeling approach provides single values for risk and hazard assessment and prioritization rather than multiple binary PBT pass/fail scores that are difficult to interpret in terms of risk and prioritization and do not address uncertainties in the data (Arnot and Mackay 2008; Zhang et al. 2010). The mass balance approach may therefore be more effective when seeking possible alternatives for chemical replacement (Lakind and Birnbaum 2010; Lavoie et al. 2010) because uncertainty in chemical information, particularly emission rates, can be included and the results compared.

An internal exposure metric may be the most biologically and toxicologically relevant because the site of toxic action is typically located inside the body. Calculated human concentrations can be integrated with toxicity data such as tissue concentrations, providing direct linkages to high throughput toxicity test data for screening-level risk assessment (Judson et al. 2011). Biomonitoring data, such as those obtained through National Health and Nutrition Examination Survey (NHANES), are valuable sources of exposure information for science and regulatory purposes. Exposure models that include human concentrations can more fully *a*) maximize the value of biomonitoring data by quantitatively linking emission rates and exposure pathways to internal doses (Woodruff et al. 2011), and *b*) complete model evaluations through comparisons with biomonitoring data (Cowan-Ellsberry et al. 2009), that is, hypothesis testing. It should be recognized, however, that internal exposure estimates such as *C* require chemical-specific information on absorption and clearance from the body (i.e., metabolic biotransformation). Most notably, the biotransformation *HL* parameter generally contributes a substantial amount of uncertainty in the calculated human concentrations.

We emphasize that the screening results for “actual” exposures should be interpreted with some skepticism. Because of the complex issues in estimating *E*_A_, a high level of error is expected and these errors in some cases may be beyond what we have attempted to quantify. In particular, the EU TGD emission scenarios are considered “realistic worst case” for screening-level objectives; therefore, the “actual” exposure estimates are expected to be conservative, particularly for high production volume chemicals that are used as intermediates or not released to the environment following the assumed emission scenario estimates.

The unit emission-based metrics are useful when few or no emissions data are available. The screening results using *C*_U_ and *iF* provide guidance for exposure hazard potential and the relative information can be useful for benchmarking chemicals before considering industrial production (Cowan-Ellsberry et al. 2009). There are many chemicals in the case study that are consistently ranked relatively high or relatively low by all exposure metrics; however, correlations in ranking results are not strong, confirming that the metrics provide different information. In summary, there are trade-offs in using different metrics for far-field human exposure assessment; the greater discriminatory power of the metrics with more toxicologically relevant (internal doses) and more “realistic” exposure assessment objectives (i.e., using estimates of actual emission rates rather than unit emission rates) are accompanied by greater uncertainty.

*Addressing uncertainty*. Different types of uncertainty exist (Finkel 1990). We consider estimates of the uncertainty in chemical information only and do not consider variability related to numerous physical and biological processes. The results are a function of the chemical properties, the model, simulation assumptions (e.g., no dissociation), parameters used to characterize the environmental and human conditions in the model and the professional judgment applied to quantify uncertainty in chemical information (input parameters). Clearly, changes to the model or simulation assumptions will result in some changes in the relative ranking and the large-scale uncertainty analysis. For example, the representative human dietary preferences in the model are constant, and the assumed mode-of-entry information is based on default EU TGD emissions scenarios. Because of a lack of data and models for parameterizing and simulating far-field human exposures to diverse types of ionogenic chemicals, we did not include dissociation in this case study. The implications of this assumption are unquantifiable errors in the exposure estimates for chemicals that are appreciably dissociated at environmental and physiological pH. There is a general need to improve measurements, models and monitoring data for ionogenic chemicals. High throughput screening-level “near-field” human exposure models are not currently available but are required to better screen human exposures and expand the mass balance framework to more fully quantify source-to-dose relationships.

Approximately 33% of the chemicals have predicted partitioning properties outside of the range of current measurements. This suggests that a large number of chemicals that are being evaluated may have highly uncertain predicted properties; however, there is no regulatory guidance for assessing such chemicals and for addressing the uncertainty of these predictions. It is stressed that the *Cf*s we selected here to address uncertainty in chemical information (model input parameters) necessarily required professional judgment because of substantial data gaps in measured and predicted chemical information. Because of the data gaps and the current screening-level approach, some codependence in uncertainty in model input parameters may occur in some instances; however, given the overall limitations in obtaining robust uncertainty estimates for all input parameters and the generally large uncertainties in model output, the issue of codependence is best addressed at higher tier assessments or when better data are available to characterize uncertainty. We expect uncertainty estimates for information used in chemical evaluations to evolve with the availability of more measured data and improved measurement techniques, better QSA(P)R models, and experience.

## Conclusions

This study has provided a quantitative perspective on the uncertainty in exposure data to better address uncertainty in screening-level exposure and risk assessment. The results indicate that more measured data and models are needed for environmental degradation *HL*s and biotransformation *HL*s. If uncertainties in other chemical properties are reduced through refined property measurement and QSA(P)R development, chemical assessments will still be highly uncertain because of the prevailing contribution to uncertainty of *E*_A_. Although continued academic research may result in some modest improvements in estimating *E*_A_ and the associated uncertainty in this parameter, any substantial improvements to reduce this key source of uncertainty in exposure and risk assessment will ostensibly require coordinated efforts with the chemical regulatory, manufacturing, and use communities. It must be recognized that the same general level of uncertainty in screening and prioritization results shown in this case study can be expected using other methods (i.e., PBT methods or other mass balance models). Improved information on chemical production and usage and addressing key sources of uncertainty such as biotransformation and environmental degradation *HL*s and continued model refinement are required to improve chemical screening and prioritization efforts.

## Supplemental Material

(147 KB) PDF
