# Supplementary material for: Prioritizing Chemicals and Data Requirements for Screening-Level Exposure and Risk Assessment
Source: Environ Health Perspect. 2012 Sep 20;120(11):1565–70. doi: 10.1289/ehp.1205355 (PMC3556628; doi:10.1289/ehp.1205355)
Supplement: (147 KB) PDF [file ehp.1205355.s001.pdf]

Supplemental Material:

Prioritizing Chemicals and Data Requirements for Screening-Level Exposure and Risk Assessment

Jon A. Arnot [1], Trevor N. Brown [1], Frank Wania [1], Knut Breivik [2, 3] and Michael S. McLachlan [4]

[1] Department of Physical and Environmental Sciences, University of Toronto Scarborough, Toronto, ON, Canada
[2] Norwegian Institute for Air Research, Kjeller, Norway
[3] Department of Chemistry, University of Oslo, Oslo, Norway
[4] Department of Applied Environmental Science (ITM), Stockholm University, Stockholm, Sweden

Table of Contents for Supplemental Material

1. Case Study Chemicals S-2

2. Model Parameterization S-2

2.1 Partitioning properties S-2

2.2 Degradation half-lives S-4

2.3 Emission estimates S-7

Supplemental Material Table S-1 S-9

3. References for Supplemental Material S-10

A version of RAIDAR Ver.2.00 is freely available at www.arnotresearch.com

1. Case Study Chemicals

We compiled a database of approximately 15,800 organic chemicals including substances with unique CAS Registration Numbers and any reported production from five national and international production volume lists (see Section 2.3 Emission estimates). This initial list includes neutral organics, organic acids and bases and organic salts. A necessary simplification was to simulate exposures based on the properties of the neutral forms of the chemicals in the database. Simplified Molecular Input Line Entry System (SMILES) notations (Weininger 1988) for organic salts were modified by removing the salt moiety, thus providing structural information (*i.e.* SMILES) for the neutral form of the molecule. Following this approach, chemical substances on the list can have different CAS numbers but the same "stripped" SMILES notation and thus modelled properties. A distinction is made between the number of chemical substances on the list and the number of unique chemical structures used in the case study exposure simulations (n=12,619). Production volumes are added for chemicals with the same "stripped" SMILES notation.

2. Model Parameterization

2.1 Partitioning properties

This section describes the methods for selecting partitioning property data and screening-level uncertainty estimates (confidence factors; *Cf*). We used the free and publicly available EPI Suite™ Ver. 4.1 (U.S. EPA 2011) experimental database and QSA(P)R models and selected measured values preferentially over modelled estimates. There are recognized uncertainties measuring and predicting chemical properties. EPI Suite™ guidance states that there is no universally accepted definition for model domain and suggests users consider the range of molecular weight in the training sets and other factors. There are limits to current measurement techniques for chemical properties (*e.g.* (OECD 2002) and there are technical challenges associated with obtaining accurate measurements near the analytical limits. Therefore, in general terms we assumed that "extreme" or "outlier" property measurements near, or beyond, the analytical limits are more uncertain than other measurements. While it is plausible that QSA(P)R models can make predictions outside of the measured property range used to develop and test the models, we considered such predictions to be "out of the model domain" and thus "highly uncertain" for this case study. We considered the extent to which chemical properties have been measured to select lower and upper bound property values to address "highly uncertain" physical-chemical property estimates (*e.g.* (Thomas 1990)). For example, as outlined below, there are relatively more octanol-water partition coefficient (K_{OW}) measurements than Henry's law constant (H) measurements, thus the assignment of uncertainty to K_{OW} is generally lower than the assignment of uncertainty to H. We identified predicted property values that were beyond the selected lower and upper bound values. We counted the occurrence of these "outlier" values and replaced them with selected lower ("minima") and upper ("maxima") bound property values recognizing that the uncertainty in these predictions may be substantial and not adequately characterized

by the current approach. We used the EPI Suite™ QSA(P)R training and testing statistics to guide the judgement and selection of *Cf*s.

Octanol-water partition coefficient. The EPI Suite™ Ver. 4.1 database includes 1,911 K_{OW} (unitless) measurements for the 12,619 unique SMILES (*i.e.*15%). The 0.5 and 99.5 percentiles for the measured K_{OW} values in this dataset are $10^{-3.7}$ and $10^{8.6}$. We selected lower and upper bound measurement limits for K_{OW} of 10^{-4} and 10^{9} as the "domain" for K_{OW} for this case study. We used the standard deviation of the model predictions against the model training dataset to provide some general guidance for overall measurement error and uncertainty. The log 10 standard deviation of the KOWWIN model against the measured training set is 0.217. Following Slob (Slob 1994) and Equation 4 in the main text, this log 10 standard deviation corresponds to a *Cf* of about 3; therefore, substances with measured K_{OW} values within the defined domain were assigned a screening-level *Cf* of 3. Measured K_{OW}s that are outside of the selected domain were considered measurement outliers (*n*=14) and were replaced with the lower and upper bound domain values, respectively, and assigned *Cf*s of 10.

Validation of the EPI Suite™ KOWWIN model (*n*=10,946 substances) with an external test set shows that 95.6% of the predicted values are within ±1 log unit of the measured values (U.S. EPA 2011). The log 10 standard deviation from the test set is 0.479, corresponding to a *Cf* of about 9 (*i.e.* using Equation 4). EPI Suite™ predicted K_{OW} values within the range for 10^{-4} to 10^{9} (*i.e.* "considered within the model domain") were therefore assigned a screening-level *Cf* of 10. We considered predicted K_{OW} values $< 10^{-4}$ and $> 10^{9}$ highly uncertain (*i.e.* "outside the model domain") and we replaced these values with the lower and upper bound values, respectively. Predicted K_{OW} values outside of the defined domain were assigned screening-level *Cf*s of 30 (*i.e.* *n*=1,253 or about 10% of the chemicals in the dataset).

Henry's law constant. The EPI Suite™ Ver. 4.1 database includes 1,255 Henry's law constant (*H*; $Pa.m^3/mol$) measurements (at 18-25 °C) for the 12,619 unique SMILES (*i.e.*10%). Many of the values reported in the measured database are calculated from measured water solubility (S_W; mol/m^3) and vapor pressure (P; Pa) data as $H = P/S_W$. The 0.5 and 99.5 percentiles for the measured *H* values in this dataset are 4.8×10^{-9} and 1.1×10^{6} $Pa.m^3/mol$. When converted to dimensionless air-water partition coefficients (K_{AW}) by division by the gas constant *R* (8.314 $Pa.m^3/(mol.K)$) and temperature *T* (298 K), these percentiles correspond to K_{AW} values of approximately 10^{-12} and 10^{3}. Therefore, we selected lower and upper bound limits for K_{AW} of 10^{-12} and 10^{3} as the "domain" for this case study. The log 10 standard deviations of the HENRYWIN models (*i.e.* both the "bond method" and the "group method") from their corresponding measured training sets are both 0.40 corresponding with *Cf*s of about 6 (*i.e.* using Equation 4 in the main text). Therefore, we assumed a *Cf* of 6 to characterize the uncertainty in measured *H* values (*n*=1,255). We replaced *H* measurements outside of the defined domain (*n*=17) with the selected lower and upper bound limits accordingly and assigned *Cf*s of 10.

We calculated *H* from measured P (*n*=455) and the average of the two water solubility QSA(P)R predictions in EPI Suite™ (*i.e.* WSKOWWIN and WATERNT) and

from measured S_W (n=702) using estimated P. We considered these 1,157 "semi-empirical" estimates of H since the estimates include 1 of 2 potentially measured values. Standard deviations (log 10) from model test sets for P and S_W are about 1 corresponding to *Cf*s of about 90 (*i.e.* using Equation 4 in the main text). The "bond method" in HENRYWIN has slightly better test statistics and has been shown to outperform the "group method" in an independent evaluation (Altschuh et al. 1999). Therefore, we used the "bond method" predictions for the remaining 10,207 chemicals unless the model was not able to make a prediction in which case we calculated H from predicted P and S_W. The log 10 standard deviation of the "bond method" QSAR against the testing set (n=1,376) is 0.475 corresponding to a *Cf* of about 8.5 (*i.e.* using Equation 4 in the main text). The H and P models are shown to have decreased performance when H is low and P is low, respectively. For example, errors in QSA(P)R predictions are notably greater when $K_{AW} < 10^{-5}$. To address this, we selected and assigned two sets of *Cf*s to QSA(P)R and "semi-empirical" estimates of H when the values fall within the defined domain as follows: if $K_{AW} < 10^{3}$ or $\geq 10^{-5}$ then $Cf = 30$, if $K_{AW} < 10^{-5}$ or $> 10^{-12}$ then $Cf = 100$. For the 2,852 chemicals with model and "semi-empirical" estimated values that fall outside of the defined model domain for H (about 23% of the case study dataset), we replaced the predicted values with the minimum or maximum domain values accordingly and assigned *Cf*s of 300 to these estimated property values.

2.2 Degradation half-lives

This section describes methods for estimating degradation half-lives (*HL*; h) and *Cf*s. There are few measured data available and there are recognized challenges estimating biodegradation *HL*s for multimedia models, *e.g.* (Arnot et al. 2005; Aronson et al. 2006; Fenner et al. 2006; Howard 1985); therefore, selecting median values and characterizing uncertainty for degradation *HLs* for most environmental media requires more assumptions and professional judgment than required for partitioning properties.

Air. Primary transformation reactions between hydroxyl (OH) radicals and organic chemicals and between ozone (O_3) and olefinic/acetylenic compounds have been measured in the gas phase. We obtained measured and predicted gas phase rate constants for these reactions from the AOPWIN database and model in EPI Suite™ Ver. 4.1 (U.S. EPA 2011). For the 12,619 unique SMILES in the present study, there are 465 and 140 measured laboratory data for hydroxyl radical and ozone reactions, respectively.

The predictive models in AOPWIN are "based upon Structure-Activity Relationship methods" (U.S. EPA 2011) by Atkinson and colleagues, *e.g.* (Atkinson and Carter 1984; Atkinson 1985; Atkinson et al. 2008). For the 12,619 unique SMILES in the present case study dataset, AOPWIN gas phase predictions for OH radical and O_3 reactions were possible for 12,536 and 2,514, respectively. The AOPWIN model development and test sets are not clearly stated; however, the log 10 standard deviations for the QSARs for OH reactions are on the order of 0.21 to 0.24 and about 0.42 to 0.52 for O_3 (Meylan and Howard 1993). These uncertainty estimates correspond to *Cf*s of about 3 and 10, respectively (Slob 1994).

Chemical degradation in the atmosphere is a function of the reaction constant and the oxidant concentration. Assuming a constant oxidant concentration, a pseudo first-order reaction half-life can be calculated, i.e., *HL*=ln(2)/*k*[oxidant]; however, OH and O_3 concentrations in the lower troposphere are also uncertain, thus contributing uncertainty to the required model input parameter. We assumed median OH and O_3 concentrations of 2×10^6 molecules (radicals)/cm^3 per 12 hours of daylight and 7×10^{11} molecules/cm^3 per day, respectively, and based on the range of typical annual oxidant concentration estimates in sub-tropical and temperate latitudes of the northern hemisphere (~ 15 to 60° N) and different altitudes (regional environment has a height of 1 km) (Atkinson and Arey 2003; Bahm and Khalil 2004), we assigned *Cf*s of 10 and 30 to the OH and O_3 half-lives, respectively. Although we selected measured data preferentially over modelled rate constant estimates, there was no distinction made for the assignment of *Cf*s. For substances subject to both reactive processes (*i.e. n*~2,578), we added the half-lives as reciprocals to obtain an overall primary degradation *HL* in air. If the overall reaction *HL* was dominated by the O_3 process (*i.e.* 35% of the 2,580 substances), we applied a *Cf* of 30 otherwise we applied a *Cf* of 10. For substances that had a total reaction rate of zero or for which a *HL* could not be determined, we assumed a *HL* of 55,000 d (Mackay et al. 2006) and we applied a *Cf* of 100.

For this case study we did not consider reactions with nitrate radicals (and other possible oxidizing agents in the atmosphere) because of a lack of available QSARs for large scale screening assessments. Also, there is some uncertainty as to whether or not reactions in the atmosphere occur strictly in the gas phase or if the same rates of reaction occur for chemicals adsorbed or absorbed in or on aerosol. For these screening-level simulations we assumed that reactions only occur in the gas phase.

Water. Empirical data for environmental biodegradation *HL* data are only available for relatively few chemicals (*i.e.* some biocides and polycyclic aromatic hydrocarbons) and therefore we did not consider these data in this case study. The only free, publicly available screening model that provides environmental biodegradation *HL* information is the BioHCwin model in EPI Suite™ Ver. 4.1 (U.S. EPA 2011). The BIOWIN sub-models include significant inherent information on the relative biodegradability of chemicals derived from different sources including measured field and laboratory data and expert survey knowledge; however, these models do not provide direct *HL* information for multimedia modeling. To address the need for environmental *HL*s, BIOWIN calibration methods have been suggested (Arnot et al. 2005; Aronson et al. 2006; Fenner et al. 2006).

We used a BIOWIN calibration method described elsewhere (Arnot et al. 2005) to obtain estimates of "environmentally relevant half-lives". Briefly, the raw numerical output of the BIOWIN sub-models: (i) the Ultimate Survey Model (USM), (ii) the Primary Survey Model (PSM), (iii) the BIODEG Linear Model (BIODEG), and (iv) the MITI Linear Model (MITI) were regressed against a dataset of environmental aerobic biodegradation *HL*s (Arnot et al. 2005). The environmental *HL* data used in the QSAR calibrations and model evaluations for chemicals with more than three environmental half-life measurements have *Cfs* that span from 2 to 90 with a median value of 9 (Arnot

et al. 2005). Comparing the average of these four predictions to a dataset of 118 substances with environmental half-life estimates (ranging from 1.3 to 2900 days) shows that approximately 95% of the predicted values are within 0.92 log units of the selected estimates (Arnot et al. 2005). This uncertainty of the averaged model predictions to this dataset of environmental half-lives corresponds to a *Cf* of 8. Therefore, we assumed a "de minimus" *Cf* of 10 for the biodegradation *HL*s for our case study simulations. We calculated a *Cf* for the biodegradation *HL* estimates from the log 10 standard deviation from the four different BIOWIN model predictions for each chemical and added it to the de minimus value of 10. *Cf*s based on differences in model predictions for the list of 12,619 substances ranged from 1.1 to 3200 with a median value of 3.5. The 99.5 percentile is 160 and 24 substances had *Cf*s that were greater than 300.

For hydrocarbons, we used the BioHCwin model to obtain median biodegradation *HL* estimates for 468 of the 12,619 unique structures. The log 10 standard deviation from the BioHCwin test set (n=54) is 0.34 corresponding to a *Cf* of 5. The BioHCwin model predicts extremely long, and seemingly unrealistic half-lives for certain chemicals, e.g., a terpene predicted half-life is 2×10^{10} d. A maximum degradation half-life of 3650 d was assumed for all substances with predicted values that were greater than 3650 d because the model results are relatively insensitive to values greater than 10 years. Following the same general approach for "non-hydrocarbons" outlined above, we calculated the *Cf*s using the four BIOWIN models and added these *Cf*s to the BioHCwin model baseline "de minimus" *Cf* of 5.

Biodegradation reactions were assumed to occur in the bulk water compartment, *i.e.* dissolved and particle bound. We did not consider degradation reactions resulting from hydrolysis and photolysis in the case study simulations.

Soil. We assumed an extrapolation factor of 1:2 for converting the aerobic water biodegradation half-lives to soil values, based on (Aronson et al. 2006). The *Cf*s for water were doubled for the soil compartment.

Sediment. We assumed an extrapolation factor of 1:9 for converting the aerobic water biodegradation half-lives to sediment values, based on (Aronson et al. 2006). The *Cf*s for water were increased by a factor of 3 for the sediment compartment.

Biota. The screening-level primary metabolic biotransformation half-life input requirements are grouped into two general organism classes; "fish" and "avian/mammals". The chemical-specific biotransformation half-life inputs (HL_N) in RAIDAR require values normalized to 1 kg organism (M_N). The normalized half-lives are then scaled in the RAIDAR model to biotransformation half-lives for the different representative species (HL_i) in the model that have different masses (M_i; kg) as:

$$HL_i = HL_N \times (M_i / M_N)^{0.25} \qquad \text{(S1)}$$

In vivo estimates for fish primary biotransformation *HL*s are available for 299 of the 12,619 unique structures (Arnot et al. 2008; U.S. EPA 2011). We used the BCFBAF

model in EPI Suite™ Ver. 4.1 (U.S. EPA 2011) to estimate whole body primary biotransformation half-lives for the remaining substances. We used recommended screening-level minimum whole body primary biotransformation half-lives to replace model predicted values that are shorter than the recommended values as detailed elsewhere (Arnot et al. 2009). The log 10 standard deviation of the biotransformation *HL* against the measured training set (*n*=421 substances) is 0.49 corresponding to a *Cf* of about 9. We assigned chemicals with in vivo estimates a screening-level *Cf* of 10. Half-life predictions compared against external test data (*n*=211 substances) show a log standard deviation of 0.60 for the estimation error, which suggests a *Cf* of 15 for in silico estimates. It is noted that errors in K_{OW} predictions by KOWWIN are automatically propagated into errors for biotransformation half-life since K_{OW} is an input parameter in the BCFBAF biotransformation *HL* model (Arnot et al. 2009).

To our knowledge there are no publicly available QSA(P)Rs for obtaining screening-level estimates of primary biotransformation *HL*s in birds and mammals. In this case study, we assumed the screening-level biotransformation *HL*s for birds and mammals to be equal to the biotransformation *HL*s for fish (*i.e.* another vertebrate species) on a per-body weight basis. We multiplied the *Cf* for fish by 3 as an assumed inter-species uncertainty scaling factor to obtain *Cf*s for HL_{BIO} for birds and mammals.

2.3 Emissions estimates

Actual emission rate data are basically non-existent for the vast majority of chemicals requiring evaluation (Egeghy et al. 2012). Our objective was to obtain some preliminary estimates of production, use and emissions for intentionally produced commercial chemicals. We obtained quantity (Q) estimates following the approach and data outlined earlier (Brown and Wania 2008). Briefly, we obtained production data from five national and international production volume lists including: (i) the Canadian Domestic Substances List (Environment Canada 2006), (ii) the U.S. EPA's High Production Volume (HPV) Challenge Program Inventory Update Rule (U.S. EPA 2006), (iii) the Japanese HPV list (METI 2008), (iv) the European Chemical Substances Information System (ESIS) (ESIS), and (v) the OECD's list of HPV chemicals (OECD 2004). It must be recognized that there may be overlaps in the data and that some sources of information such as the Canadian DSL (ca. 1985) are severely outdated. Another major complicating factor is that the production data are typically presented as bins, rather than discrete numerical values, e.g., "100,000 to 1,000,000 t/y". In addition, the top-level bins are typically open-ended, and a large maximum production value had to be assumed (10,000,000 t/y or 10 times greater than the largest upper bin reported, from the Japanese HPV list). We summed minimum and maximum overall production volumes for substances that had the same "stripped" SMILES notation and we calculated the total geometric mean production quantity (M_{Q_T}) from the total minimum (MIN_{Q_T}) and maximum (MAX_{Q_T}) values.

Presumably the maximum emission rate can only approximate the maximum total production rate for chemicals that are intentionally released to the environment (*e.g.* certain biocides). On the other hand, the minimal emission rate can be negligible for

chemicals that are consumed entirely during production. Moving from highly uncertain production quantities to estimates of actual emission rates is challenging; however, screening-level methods have been developed. Most notably, the EU Technical Guidance Document (TGD) on Risk Assessment provides recommendations for emission scenarios that were initially developed to obtain "realistic worst-case emission scenarios" (European Commission 2003). Following the EU TGD, we estimated emissions from quantity estimates and physical-chemical property information. As outlined in Table S-1, physical-chemical properties alone are assumed to provide information on the relative release of chemical to air, water and soil. The specific emission factors listed in Table S-1 furthermore represent "default" recommended values whenever information on chemical function is lacking, lumped across three stages of the commercial life-cycle of a chemical (production, formulation and use). The use of physical-chemical properties to estimate the actual emission rate is quite limited (Fauser et al. 2010); however, chemical function information is not currently available for the vast majority of the chemicals.

The regional scale estimate for the actual chemical emission rate (E_A; kt/y) is required by the model for actual exposure estimates. The regional scale of the evaluative environment is 10^5 km^2 and the production data are from various countries and international organizations. We assumed a general population density of 50 persons/ km^2 and a population of 2.5 billion people associated with the use and release of these chemicals, and we applied a factor of 500 to obtain a regional median quantity (M_{Q_R}) from M_{Q_T} (*i.e.* a global-scale quantity). The scaling factor of 500 is a crude approximation; however, since this research is largely focused on identifying sources of uncertainty in screening-level exposure assessment and these data are not being used for actual risk assessment the scaling factor is somewhat arbitrary. The EU TGD emissions scenarios are applied to M_{Q_R} to obtain E_A and mode-of-entry (MOE) information.

According to the EU TGD "worst case emission scenarios" the percentages for actual chemical emissions from Q estimates range from 4.1 to 83.5% (see Table S-1). However, as discussed above the percentages for certain chemicals may fall outside this range. While there are undoubtedly uncertainties in the % MOE, the EU TGD emission scenarios provide some screening-level guidance for % MOE; however, they are considered too conservative in the context of the plausible range of actual emission rates. We estimated screening-level *Cf*s as $0.1 MAX_{Q\_T}$ / M_{Q_R}. This is also a conservative screening-level assumption suggesting that the maximum chemical quantity that can be released in a regional scale environment (10^5 km^2) is 10% of the M_{Q_T}, *e.g.,* representing a production facility associated with high emissions of a particular chemical to the regional environment in addition to per capita usage. The current approach does not likely adequately address the true uncertainty in the distribution for emissions, *i.e.* the true lower bound estimate is not likely captured for certain substances such as chemical production intermediates. Thus for many substances the current approach is likely very conservative. Characterizing the uncertainty in the actual emissions rate here is also very preliminary, but a necessary first step to obtain some context of the relative data gaps and uncertainties in high throughput exposure and risk assessment.

Supplementary Material Table 1. European Union Technical Guidance (TGD) emissions factors (European Commission 2003) used in the case study to estimate chemical mode of entry and the fraction of quantity that is actually released to the environment.

Fraction released to air

Vap\Sol	<100 mg/L	100-1000	>=1000
<1 Pa	0.004	0.003	0.003
1-10	0.004	0.003	0.003
10-100	0.016	0.007	0.006
100-1000	0.120	0.070	0.021
1000-10000	0.575	0.175	0.085
>10000	0.825	0.575	0.175

Fraction released to wastewater

Vap\Sol	<100 mg/L	100-1000	>=1000
<1 Pa	0.11	0.51	0.76
1-10	0.11	0.51	0.76
10-100	0.02	0.11	0.51
100-1000	0.01	0.02	0.11
1000-10000	0.01	0.01	0.02
>10000	0.01	0.01	0.01

Fraction released to soil

Vap\Sol	<100 mg/L	100-1000	>=1000
<1 Pa	0.0092	0.0027	0.0004
1-10	0.0092	0.0027	0.0004
10-100	0.0051	0.0011	0.0004
100-1000	0.0011	0.0007	0.0002
1000-10000	0.0004	0.0002	0.0002
>10000	0.0002	0.0002	0.0002

Fraction released to the environment (all media)

Vap\Sol	<100 mg/L	100-1000	>=1000
<1 Pa	0.12	0.51	0.76
1-10	0.12	0.51	0.76
10-100	0.04	0.11	0.51
100-1000	0.13	0.09	0.13
1000-10000	0.58	0.18	0.10
>10000	0.83	0.58	0.18

3. References for Supplemental Material

Altschuh J, Bruggemann R, Santl H, Eichinger G, Piringer OG. 1999. Henry's law constants for a diverse set of organic chemicals: Experimental determination and comparison of estimation methods. Chemosphere 39:1871-1887.

Arnot JA, Gouin T, Mackay D. 2005. Practical Methods for Estimating Environmental Biodegradation Rates. Report for Environment Canada. CEMN2005003. Peterborough, ON: Canadian Environmental Modelling Network, Trent University.

Arnot JA, Mackay D, Parkerton TF, Bonnell M. 2008. A database of fish biotransformation rates for organic chemicals. Environ Toxicol Chem 27:2263-2270.

Arnot JA, Meylan W, Tunkel J, Howard PH, Mackay D, Bonnell M, et al. 2009. A quantitative structure-activity relationship for predicting metabolic biotransformation rates for organic chemicals in fish. Environ Toxicol Chem 28:1168-1177.

Aronson D, Boethling R, Howard P, Stiteler W. 2006. Estimating biodegradation half-lives for use in chemical screening. Chemosphere 63:1953-1960.

Atkinson R, Carter WPL. 1984. Kinetics and mechanisms of the gas-phase reactions of ozone with organic compounds under atmospheric conditions. Chem Rev 84:437-470.

Atkinson R. 1985. Kinetics and mechanisms of the gas-phase reactions of the hydroxyl radical with organic compounds under atmospheric conditions. Chem Rev 85:69-201.

Atkinson R, Arey J. 2003. Atmospheric degradation of volatile organic compounds. Chem Rev 103:4605-4638.

Atkinson R, Arey J, Aschmann SM. 2008. Atmospheric chemistry of alkanes: Review and recent developments. Atmos Environ 42:5859-5871.

Bahm K, Khalil MAK. 2004. A new model of tropospheric hydroxyl radical concentrations. Chemosphere 54:143-166.

Brown TN, Wania F. 2008. Screening chemicals for the potential to be persistent organic pollutants: A case study of Arctic contaminants. Environ Sci Technol 42:5202-5209.

Egeghy PP, Judson R, Gangwal S, Mosher S, Smith D, Vail J, et al. 2012. The exposure data landscape for manufactured chemicals. Sci Total Environ 414:159-166.

Environment Canada. 2006. Existing Substances Program at Environment Canada (CD-ROM). Ecological Categorization of Substances on the Domestic Substances List (DSL). Available: http://www.ec.gc.ca/substances/ese/eng/dsl/cat_index.cfm. [accessed 15 July 2007].

ESIS. European Chemical Substances Information System. Ispra, Italy: European Commission. Joint Research Centre. Available: http://esis.jrc.ec.europa.eu/ [accessed April 2007].

European Commission. 2003. Technical Guidance Document on Risk Assessment in support of Commission Directive 93/67/EEC on Risk Assessment for New Notified Substances, Commission Regulation (EC) No 1488/94 on Risk Assessment for Existing Substances, Directive 98/8/EC of the European Parliament and of the Council Concerning the Placing of Biocidal Products on the Market. Ispra, Italy: Joint Research Centre, Institute for Health and Consumer Protection, European Chemicals Bureau.

Fauser P, Thomsen M, Pistocchi A, Sanderson H. 2010. Using multiple regression in estimating (semi) VOC emissions and concentrations at the European scale. Atmos Poll Res 1:132-140.

Fenner K, Canonica S, Escher BI, Gasser L, Spycher S, Tulp HC. 2006. Developing methods to predict chemical fate and effect endpoints for use within REACH. Chimia 60:683-690.

Howard PH. 1985. Determining "real world" biodegradation rates. Environ Toxicol Chem 4:129-130.

Mackay D, Shiu WY, Ma KC, Lee SC. 2006. Handbook of Physical-Chemical Properties and Environmental Fate for Organic Chemicals, Second Edition. Vol I-IV.: CRC Press.

METI. 2008. Japan HPV Challenge Program. Tokyo, Japan: Ministry of Economy, Trade and Industry (METI).

Meylan WM, Howard PH. 1993. Computer estimation of the atmospheric gas-phase reaction rate of organic compounds with hydroxyl radicals and ozone. Chemosphere 26:2293-2299.

OECD. 2002. Proposal for updating guideline 104: Vapour pressure. (OECD Guidelines for the Testing of Chemicals). Paris: Organization for Economic Co-operation and Development.

OECD. 2004. The 2004 OECD list of high production volume chemicals. Paris, France: Organization for Economic Cooperation and Development (OECD), Environment Directorate.

Slob W. 1994. Uncertainty analysis in multiplicative models. Risk Analysis 14:571-576.

Thomas R. 1990. Volatilization from water. In: Handbook of Chemical Property Estimation Methods Environmental Behavior of Organic Chemicals, (Lyman WJ, Reehl,W.F., and Rosenblatt, D.H., ed). Washington DC: American Chemical Society.

U.S. EPA. 2006. High production volume (HPV) challenge program. The HPV voluntary challenge chemical list.

U.S. EPA. 2011. Estimation Programs Interface (EPI) Suite™ for Microsoft® Windows, Ver. 4.1., Part Released October, 2011. Washington, D.C.: U. S. Environmental Protection Agency.

Weininger D. 1988. SMILES, a chemical language and information-system. 1. Introduction to methodology and encoding rules. J Chem Inf Comp Sci 28:31-36.
